# Comparison of a Genotype 1 and a Genotype 2 Macaque Foamy Virus *env* Gene Indicates Distinct Infectivity and Cell-Cell Fusion but Similar Tropism and Restriction of Cell Entry by Interferon-Induced Transmembrane Proteins

**DOI:** 10.3390/v15020262

**Published:** 2023-01-17

**Authors:** Thomas Fricke, Sarah Schlagowski, Shanchuan Liu, Xiaoliang Yang, Uwe Fiebig, Artur Kaul, Armin Ensser, Alexander S. Hahn

**Affiliations:** 1 Junior Research Group Herpesviruses, German Primate Center—Leibniz-Institute for Primate Research, 37077 Göttingen, Germany; 2 Robert Koch Institute, Sexually Transmitted Bacterial Infections (STI) and HIV, 13353 Berlin, Germany; 3 Infection Biology, German Primate Center—Leibniz-Institute for Primate Research, 37077 Göttingen, Germany; 4 Institute for Clinical and Molecular Virology, Friedrich-Alexander-Universität Erlangen-Nürnberg, 91054 Erlangen, Germany

**Keywords:** foamy virus, interferon-induced transmembrane proteins, envelope, virus entry, fusion

## Abstract

Foamy viruses (FVs) are naturally found in many different animals and also in primates with the notable exception of humans, but zoonotic infections are common. In several species, two different envelope (*env*) gene sequence clades or genotypes exist. We constructed a simian FV (SFV) clone containing a reporter gene cassette. In this background, we compared the *env* genes of the SFVmmu-DPZ9524 (genotype 1) and of the SFVmmu_R289hybAGM (genotype 2) isolates. SFVmmu_R289hybAGM *env*-driven infection was largely resistant to neutralization by SFVmmu-DPZ9524-neutralizing sera. While SFVmmu_R289hybAGM *env* consistently effected higher infectivity and cell-cell fusion, we found no differences in the cell tropism conferred by either *env* across a range of different cells. Infection by both viruses was weakly and non-significantly enhanced by simultaneous knockout of interferon-induced transmembrane proteins (IFITMs) 1, 2, and 3 in A549 cells, irrespective of prior interferon stimulation. Infection was modestly reduced by recombinant overexpression of IFITM3, suggesting that the SFV entry step might be weakly restricted by IFITM3 under some conditions. Overall, our results suggest that the different *env* gene clades in macaque foamy viruses induce genotype-specific neutralizing antibodies without exhibiting overt differences in cell tropism, but individual *env* genes may differ significantly with regard to fitness.

## 1. Introduction

Foamy viruses are an ancient family of retroviruses that are found in many mammals and higher primates with which they co-speciate [1,2,3], with the notable exception of humans [4]. Endogenous foamy viruses are present in coelacanth [5], amphibia [6], fish [7], birds and serpentines [8]. SFVs have demonstrated a potential for zoonotic infection in Africa [9,10,11] and also in Asia [12,13]. Recently, zoonotic infections with gorilla SFV were found to be associated with hematological abnormalities and altered biochemical markers [9]. In addition, foamy viruses represent potential vector platforms for gene therapy [14,15,16,17] or oncolytic therapy [18,19], which warrants study of their cell entry mechanisms.

In several species, including in macaques, where multiple isolates or sequences have been obtained, there are at least two different sequence groups or clades that differ in the *env* gene [20,21,22] and most notably in a small region of the Env protein that has been proposed to act as a receptor binding domain (RBD) [23]. Three serotypes were reported to exist for feline foamy virus (FFV) [24], and different genotypes in the env locus have been characterized and were shown to correspond to these serotypes [25,26]. We recently isolated a foamy virus of rhesus macaques with a genotype 1 *env* gene, SFVmmu-DPZ9524 [20], and some time before an isolate, SFVmmu-R289hybAGM, from a rhesus monkey with a genotype 2 *env* was reported [27].

During infection with the human immunodeficiency virus, different *env* species develop that differ in coreceptor usage and cell tropism [28,29]. We were interested in whether the two envelope genotypes of rhesus macaque foamy viruses differ only in their neutralization sensitivity to antibodies elicited by viruses from their own or the respective other genotype but not in their functionality, or whether the different *env* variants confer functional differences such as altered cell tropism, which could suggest different receptor tropism or altered sensitivity to cellular restriction factors. Here, in particular, the interferon-induced transmembrane proteins (IFITM) 1-3, which restrict enveloped viruses dependent on their pathway of entry, among other determinants, at the membrane fusion step [30,31,32,33], are of interest. For FFV, human IFITMs were shown to inhibit FFV at a late stage of infection [34], hinting at additional antiviral mechanisms.

To facilitate analysis of infection, we constructed a YFP reporter virus SFV based on the SFVmmu-DPZ9524 genotype 1 isolate by replacing a part of the 3′ LTR U3 region with a YFP expression cassette. In this background, we replaced the genotype 1 *env* gene with that of the genotype 2 isolate to compare the functionality of these two *env* genes. These constructs were then analyzed with regard to susceptibility to neutralization, cell tropism, specific infectivity, and restriction by IFITMs 1-3.

## 2. Materials and Methods

### 2.1. Construction of the SFVmmu-DPZ9524 Infectious Clone

As described previously, DNA amplified from low passage virus stocks [20] was used to clone fragments of *SFVmmu-DPZ9524* into the backbone of pNCS-mNeonGreen (Allele Biotechnology, San Diego, CA, USA), replacing the mNeonGreen open reading frame. Fragments were assembled using overlapping PCR products and Gibson assembly, and checked by Sanger sequencing. Non-synonymous mutations were changed back to the reference sequence (MG051205.1), except for A to G in gag, resulting in the exchange of serine 248 to glycine as glycine was also found in a number of other foamy virus gag sequences at that position. There was an uncommonly high rate of nucleotide exchanges in the gag gene, which raises the possibility that the original isolate actually contained two viruses with slightly different gag sequences, which has been described for macaques [35]. A more trivial explanation might be that the original Illumina sequencing struggled with the quite repetitive sequence with high GC content, as this region had comparatively low read coverage, in keeping with our inability to find proof of these sequence variations by PCR. As we were only able to successfully isolate clones containing these nucleotide exchanges, we decided to proceed using them. Finally, using a clone ranging from nt1606 to the 3′ end, we amplified the LTR using primers DPZ9524 1-22 forward T7oh (TAATACGACTCACTATAGGGTGTGGCAGGCAGCCACTAAATG) and DPZ9524 1599-1638 LTR rev PBS oh (TTGGGCGCCAATTGTCATGGAATATTGTATATTGATTATC), amplified the rest of the genome and the vector using primers DPZ9524 1618-1642 forward (TCCATGACAATTGGCGCCCAACGTG) and T7 reverse (CCCTATAGTGAGTCGTATTAATTTCG) and used Gibson assembly to assemble the infectious clone AX512 pSFVmmu-DPZ9524_1. Transfection of this clone resulted in visible formation of a cytopathic effect that could be transferred, indicative of replication. The plasmid was sequenced by Next-Generation Sequencing. For library preparation, 50 ng of purified DNA and the Nextera DNA Library Preparation Kit (FC-131.1024, Illumina, San Diego, CA, USA) were used. Tagmented DNA was amplified with specific dual index primers (Integrated DNA Technologies, Coralville, IA, USA) using the NEBNext^®^ HiFi 2 × PCR Master Mix (M0541, NEB) according to the manufacturer’s protocols. The libraries were cleaned up with AMPure XP beads (A63881, Beckman Coulter, Brea, CA, USA) and quantified using the Qubit dsDNA HS Assay Kit (Q32851, Thermo Fisher Scientific, Waltham, MA, USA). The libraries were analyzed by paired-end next-generation sequencing using a 150 cycle MiSeq reagent kit v3 (forward read 90 cycles, reverse read 60 cycles) on a MiSeq™ Instrument (Illumina, San Diego, CA, USA). The sequences were analyzed with CLC Genomics Workbench 20 (Qiagen Aarhus A/S, Aarhus, Denmark). Plasmids were fully covered with a Median Coverage between 564 and 1447 (minimum coverage 282, maximum coverage 2264). We discovered a 11nt insertion compared to the original MG051205.1 sequence; this was very likely a sequence assembly error in MG051205.1, as this sequence stretch is present in all related sequences. After next generation sequencing of the final construct, an additional mutation in the bet open reading frame, C to T, resulting in mutation of His283Tyr, was discovered. This conservative mutation was not corrected as the planned study was aimed at analyzing the function of Env and not of Bet. A summary of all SNPs in comparison to Genbank MG051205.1 is provided in Table S1.

### 2.2. Construction of SFV Reporter Viruses

An expression cassette driven by the immediate early promoter of the human cytomegalovirus (CMV, CMVie) was amplified from RRV-YFP [36] (MN488839.2) using primers CMVie for BetSTOP OH.

(GTGATTCTTCAGATGAAGATTAAATTAATAGTAATCAATTACGGG) and mNeon rev Delta 3′LTR oh (GTCATCAGGAGCTAATTTTACTTGTACAGCTCGTCC) and assembled together with the foamy virus backbone amplified from a construct lacking the 5′ LTR (pNCS-Foamy9524-4167-3primeEND) using primers Bet before STOP rev (ATCTTCATCTGAAGAATCACTAGAGG) and Delta 3′LTR for (TAAAATTAGCTCCTGATGACTCACG) by Gibson assembly. This construct was amplified using primers DPZ9524 9959:9983 for (TGGGAAGATCAAGAAGAATTAAGAG) and AmpQas and assembled together with the other part of the genome amplified from the full-length infectious clone AX512 pSFVmmu-DPZ9524_1 using primers DPZ9524 9958:9982 rev (TCTTAATTCTTCTTGATCTTCCCAG) and AmpQs, resulting in AX571 pSFVmmu-DPZ9524_1_CMVie-YFP.

The *env* gene of AX571 pSFVmmu-DPZ9524_1_CMVie-YFP was exchanged with that of SFVmmu_R289hybAGM by Gibson Assembly. SFVmmu_R289hybAGM *env* was amplified from cloned proviral DNA using primers 289 env for 9524oh (ACA ATGGCACCTCCAATGAC) and DPZ9524 9958:9982 rev (TCTTAATTCTTCTTGATCTTCCCAG) and joined with the AX571 pSFVmmu-DPZ9524_1_CMVie-YFP backbone, which was amplified using primers DPZ9524 9959:9983 for (TGGGAAGATCAAGAAGAATTAAGAG) and DPZ9524 7006:7027 rev (GTCATTGGAGGTGCCATTGTTC), to generate AX585 pSFVmmu-DPZ9524_1_ R289hybAGMenv_CMVie-YFP. We noticed a sequence variation in the *env* gene of our SFVmmu_R289hybAGM clone resulting in a change of amino acid 594 from Y to N in comparison to the deposited amino acid sequence (AFA44810.1). As all other sequences identified by BLAST [37] (blastp, expect value 0.05, hitlist size 100, gapcosts 11/1, matrix BLOSUM62, low complexity filter, filter string L, genetic code 1, window size 40, threshold 21, composition-based stats 2, database Jul 22, 2022 2:52 AM) from the genotype 2 clade, even from feline foamy virus, encoded N at this position, we regarded N as an at least highly plausible variant and Y as a potential sequencing artifact and did not change the sequence back from N to Y at this position.

Env expression plasmids in pcDNA6V5his (Invitrogen, Waltham, MA, USA) were constructed by GibsonAssembly. The vector backbone was amplified using primers pcDNA6 XbaI V5 for (GTCTAGAGGGCCCTTCGAAGGTAAG) and pcDNA6 KpnI ATG rev (CATGGTACCAAGCTTAAGTTTAAAC). The respective Env coding sequences were amplified from the infectious clones using genotype-specific forward primers DPZ9524 env pcDNA6 KpnI for (AACTTAAGCTTGGTACCATGGCACCTCCAATGACCTTGG) and R289hyAGM env pcDNA6 KpnI for (AACTTAAGCTTGGTACCATGGCACCTCCAATGACTTTGG) together with the reverse primer SFV env w/oSTOP rev XbaI V5 oh (CTTACCTTCGAAGGGCCCTCTAGAATTCTTCTTGATCTTCCCAGGAAG). Other plasmids that were used for this study are described in Table 1.

### 2.3. Design and Visualization

Visualization of the genome structure was performed using SnapGene Viewer (version 6.1, www.snapgene.com) and Inkscape (version 1.1, The Inkscape Project). Sequence alignment and visualization of the Env proteins was performed using MacVector (version 18.2.5, MacVector, Inc. Cary, NC, USA) and its implementation of ClustalW using the default settings.

### 2.4. Cell Culture

All cell lines in this study (Table 2) were incubated at 37 °C and 5% CO_2_ and cultured in Dulbecco’s modified Eagle medium, high glucose, GlutaMAX, 25 mM HEPES (Thermo Fisher Scientific, Waltham, MA, USA) supplemented with 10% fetal calf serum (FCS; Thermo Fisher Scientific, Waltham, MA, USA) and 50 μg/mL gentamicin (PAN-Biotech, Aidenbach, Germany) (D10) except for HUVEC, which were maintained in standard Endothelial Cell Growth Medium 2 (PromoCell, Heidelberg, Germany), and BJAB and MDA-MB-231 cells, which were maintained in RPMI (Thermo Fisher Scientific, Waltham, MA, USA) supplemented with 10% FCS and 50 μg/mL gentamicin. Interferon-alpha (IFN-α) treatment was performed by supplementing the respective culture medium with IFN-α 2b (Sigma; 5000 U/mL). For seeding and subculturing of cells, the medium was removed, the cells were washed with phosphate-buffered saline (PBS; PAN-Biotech, Aidenbach, Germany) and detached with trypsin (PAN-Biotech, Aidenbach, Germany). All transfections were performed using polyethylenimine (PEI; Polysciences) at a 1:3 ratio (µg DNA/µg PEI) mixed in Opti-MEM (Thermo Fisher Scientific, Waltham, MA, USA).

### 2.5. Western Blot

For detection of IFITM expression, the cells were treated with or without IFN-α (5000 U/mL) for 16 h. Thereafter, the cells were harvested and subjected to Western blot analysis. SDS polyacrylamide electrophoresis (PAGE) was performed using 8–16% gradient gels (Thermo Fisher Scientific, Dreieich, Germany). Western blotting was performed as described previously [43] using the respective antibodies (Table 3). Expression of other proteins was analyzed accordingly.

### 2.6. Fusion Assay

On day one, 293T target cells were transfected in a 6-well plate overnight with 0.5 μg plasmid encoding a Gal4 response element driven TurboGFP-luciferase reporter (Gal4-TurboGFP-Luciferase). The 293T effector cells were transfected in a 6-well plate overnight either with 1 µg empty vector pNCS-mNeonGreen or AX571 SFVmmu-AX585 DPZ9524_1_CMVie-YFP (ST1) or DPZ9524_1_ R289hybAGMenv_CMVie-YFP (ST2) and 0.5 μg VP16-Gal4 transactivator plasmid. On day two, 16 h after transfection, the medium on the cells was completely removed and exchanged with fresh medium. Twenty-four hours after transfection, the effector cells were trypsinized and seeded in 96-well plates at 50,000 cells/well. On day three, the target cells were trypsinized and added to the effector cells. After 48 h, the cells were lysed in 65 μL 1× Luciferase Cell culture lysis buffer (E1531, Promega, Madison, WI, USA) for 20 min at room temperature and centrifuged for 10 min at 4 °C. Fifty microliters of each cell lysate were used to measure luciferase activity using the Beetle-Juice Luciferase Assay (PJK Biotech, Kleinblittersdorf, Germany) according to manufacturer’s instructions on a Biotek Synergy 2 plate reader. Four independent experiments were performed. Each experiment was normalized to fusion signal of 293T effector cells transfected just with empty vector pNCS-mNeonGreen and VP16-Gal4 fused with 293T target cells. A paired *t*-test was performed to compare the results of the four experiments.

For testing cell-cell fusion activity of Env expressed from CMVie promoter-driven expression plasmids, A549 target cells were transduced with the lentiviral Gal4-driven TurboGFP-luciferase reporter construct as described previously [33], and were selected using 10 µg/mL of Blasticidin for three passages prior to the experiment. On 96-well, 293T cells (30,000/well) were seeded as the effector cells. After attachment, 31.25 ng of the transactivator (Gal4-VP16) plasmid were transfected together with 93.75 ng of the respective Env expression plasmid or empty vector per well. One day post transfection, A549 target cells (40,000/well) were added to 293T effector cells. The coculture was incubated for 48 h and then processed to measure luciferase activity as described above.

### 2.7. Retroviral Vectors, Foamy Viruses and Pseudotyped Lentiviral Particles

Retroviruses encoding the IFITM expression cassettes (pQCXIP backbone), lentiviruses (plentiCRISPRv2 backbone [41]) and lentiviral pseudotypes (pLenti CMV GFP Neo (657-2)) were produced by PEI-mediated transfection of 293T cells (see Table 1 for plasmids). For the production of SFV YFP reporter virus, 293T cells were transfected overnight in T175 flasks with either 15 μg AX571 pSFVmmu-DPZ9524_1_CMVie-YFP (ST1) or AX585 pDPZ9524_1_ R289hybAGMenv_CMVie-YFP (ST2). Virus-containing cell-culture supernatant was harvested after 48 h. For retrovirus production, plasmids encoding gag/pol, pMD2.G encoding VSV-G, and the respective pQCXIP-contructs were transfected (ratio 1.6:1:1.6).

For production of lentiviruses used for transduction, psPAX2 encoding gag/pol, pMD2.G encoding VSV-G (vesicular stomatitis G protein) and the respective lentiviral construct were used (ratio 2.57:1:3.57). For lentiviral pseudotypes (LP) psPAX2, pLenti CMV GFP Neo and expression plasmids for pCAGGS IAV_WSN-HA and pCAGGS IAV_WSN-NA (encoding hemagglutinin and neuraminidase of influenza A strain WSN) for IAV-LP or paMLV_env (encoding amphotropic murine leukemia virus Env protein) for MLV-LP were used (ratio 1:1.4:2.4). Viruses were harvested twice: 24–48 h and 72–96 h after transfection.

Virus-containing supernatants were passed through a 0.45-μm CA filter and frozen at −80 °C. Transduction was performed by adding retroviruses and lentiviruses to cells for 48 h. IFITM1-, IFITM2- and IFITM3-knockout cell pools were generated by CRISPR/Cas9-mediated knockout following the protocol described by Sanjana et al. [41], except that PEI transfection was used. In short, the cells intended for knockout were transduced with lentiviruses harboring the CRISPR/Cas9 gene and sgRNAs targeting IFITM1-3 (sgIFITM1/2/3-a, sgIFITM1/2/3-b) or non-targeting sgRNAs (sgNT-a, sgNT-b). Afterwards, selection was performed using 5 μg/mL puromycin (InvivoGen; pQCXIP and pLentiCRISPRv2 constructs) for at least two passages.

### 2.8. Neutralization Assay

For neutralization assays, A549 cells were seeded in 96-well plates at 50,000 cells/well. At 6 h after plating, the SFV viruses were incubated for 30 min with or without heat-inactivated (56 °C for 30 min to inactivate the complement system) rhesus macaque sera before being added to the cells. The cells were harvested 48 h post-infection by brief trypsinization, followed by the addition of one volume of 5% FCS in PBS to inhibit trypsin activity and fixed by addition of one volume of PBS supplemented with 4% formaldehyde (Carl Roth). Ten thousand cells were analyzed per sample for YFP expression on an ID7000™ Spectral Cell Analyzer flow cytometer (Sony Biotechnology, San Jose, CA, USA). Data were analyzed using ID7000™ Spectral Cell Analyzer (Sony Biotechnology). Two independent experiments were performed for both SFVs. Each experiment was normalized to cells without immune sera infected with the respective virus.

### 2.9. Infection Experiments

Cells were seeded in 96-well plates at 90% confluency 6 h prior to infection. For IFITM ko experiments, cells were treated with IFN-α (5000 U/mL) or H2O (control) for 16 h prior to infection with either SFV ST1, SFV ST2, RRV, IAV-LP or MLV-LP. Forty-eight hours post infection, cells were trypsinized, trypsin activity was inhibited by adding 5% FCS in PBS and the cells were washed and fixed with 4% methanol-free formaldehyde (Roth) in PBS. Infection was determined by detection of GFP+/YFP+ cells (expressing the respective reporter gene of the lentiviral pseudotypes or the foamy reporter viruses) using a ID7000™ Spectral Cell Analyzer flow cytometer (Sony Biotechnology, San Jose, CA, USA).; ten thousand cells were analyzed. Curve fitting of infectivity on the different cell lines was performed using the built-in exponential equation for one phase association of GraphPad Prism version 9 for Windows (GraphPad Software, La Jolla, CA, USA) based on the Poisson distribution [43]. The span was set from 0 to 100, representing 0% or 100% infected cells, respectively, resulting in the simplified function f(x) = 100 × (1 − e^−K × x^), with x representing input (virus dilution) and K representing the specific infectivity per input. The ratio between K_ST1_ and K_ST2_ was used to calculate the differences in infectivity between the viruses.

For spin-infection (spinfection), 293T cells were seeded in 96-well plates, 50 µL of virus-containing media was added to the cells, and the plate was spun for 2 h at 800 g. After 6 h, 50 µL of fresh culture medium were added, and the cells were incubated for 3 days before harvesting for analysis of infection by flow cytometry.

Images of infected or transduced cells were acquired on a Zeiss AxioVert.A1 cell culture microscope with LED fluorescence imaging.

### 2.10. Quantitative Reverse Transcription Realtime-PCR (RT-qPCR)

Viral nucleic acid was extracted from cell culture supernatants using the Viral RNA Mini kit (Qiagen, Hilden, Germany) after (or without) digestion with DNAseI (NEB) overnight, and RNA was isolated from infected cells using RNAzol and the Direct-zol RNA Miniprep kit (Zymo, Irvine, CA, USA) according to the manufacturer’s instructions. qPCR with or without reverse transcription was performed using the Sensifast OneStep Probe HiRox Kit (Bioline, Division of Meridian Bioscience, Cincinnati, OH, USA) on a StepOnePlus system (Thermo Fisher Scientific, Waltham, MA, USA ). PCR conditions were 45 °C for reverse transcription, 2 min 95 °C initial denaturation, then 50 cycles of 95 °C for 5 s followed by 60 °C for 20 s. For the detection of the *pol* gene, primers pol for (CCCAAGGTAGTTATGTGGTTCAT), pol rev (ATGTCCTTGTAGCAACTGATCC) and probe pol prb (/56-FAM/AATACCACT/ZEN/CCAAGCCTGGATGCA/3IABkFQ/) were used, for detection of the spliced Bet mRNA primer bet for (TGGGTACCAGACCCTTCA), bet rev (CTAGGATTAGCGCGACGTTT), and probe bet prb (/56-FAM/ATTAGCCTC/ZEN/GAAGGAACTCGGCTC/3IABkFQ/) were used and for the detection of the hypoxanthine phosphoribosyltransferase (HPRT) housekeeping gene, primers hprt for TGCTGAGGATTTGGAAAGGG and hprt rev ACAGAGGGCTACAATGTGATG and probe hprt prb /56-FAM/TCGAGATGT/ZEN/GATGAAGGAGATGGGAGG-3/IABkFQ/ were used (/56-FAM indicates 5′ 6-FAM (Fluorescein) modification; ZEN indicates the internal quencher proprietary to IDT, 3IABkFQ indicates 3′ IowaBlack fluorescene quencher modification). All oligonucleotides were purchased from IDT.

Relative expression was calculated using the 2^−∆Ct^ method for each sample, then normalizing to the respective control as described by Schmittgen et al [44].

### 2.11. Statistical Analysis

Statistical analysis and data visualization were performed with GraphPad PRISM version 9.3.1.

## 3. Results

### 3.1. Generation of an Infectious Clone of SFVmmu-DPZ9524

**Figure 1:**
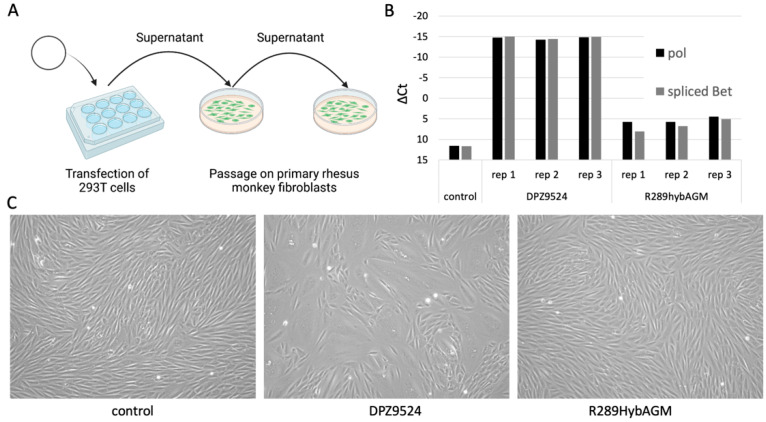
The pSFVmmu-DPZ9524_1 infectious clone yields infectious foamy virus. (**A**) After transfection of pSFVmmu-DPZ9524_1 or a proviral clone of R289hybAGM into 293T cells, cell-free supernatant from transfected cells or untransfected cells (control) was transferred to primary rhesus monkey fibroblasts. (Schematics created with BioRender.com.) (**B**) Expression of viral RNA (pol and spliced Bet mRNA) in primary rhesus monkey fibroblasts. ∆Ct = Ct_viral transcript_ − Ct_HPRT_. Ct was set to 40 cycles for negative samples (control). Values for three biological replicates (rep 1–3) and one control using supernatant from untransfected 293T cells are shown. (**C**) Representative phase contrast images of primary rhesus monkey fibroblasts after inoculation with the respective plasmid-derived foamy virus.

We first cloned the genome of SFVmmu-DPZ9524 into a plasmid backbone to obtain an infectious clone, AX512 pSFVmmu-DPZ9524_1. Transfection of this plasmid into 293T cells, transfer of the supernatant to primary rhesus monkey fibroblasts and repeat passage (Figure 1A) resulted in expression of different viral RNAs (Figure 1B) as well as readily visible cytopathic effect (CPE) and formation of syncytia (Figure 1C), all indicative of viral replication. We compared supernatants from 293T cells transfected with AX512 pSFVmmu-DPZ9524_1, with the R289hybAGM plasmid and untransfected 293T supernatant in repeat passage experiments. Passaging of pSFVmmu-DPZ9524_1-derived virus resulted in stronger CPE and higher expression of both the pol RNA fragment and also of the spliced Bet transcript, as compared to passaging of virus derived from the R289hybAGM clone, at least under these conditions (Figure 1B,C).

### 3.2. Generation of a SFV Reporter Virus with Genotype 1 and Genotype 2 env

**Figure 2:**
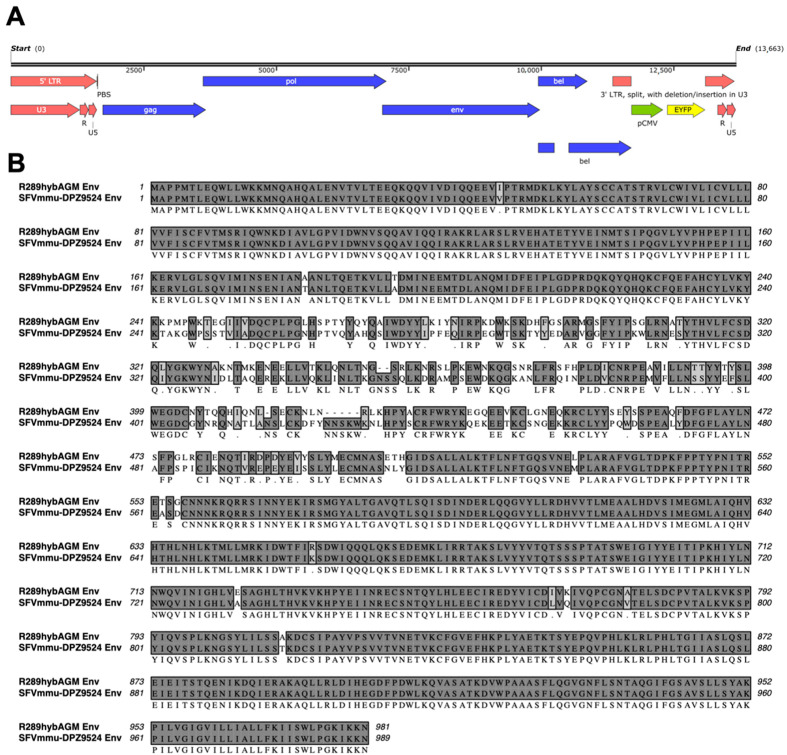
Generation and characterization of SFV reporter viruses with genotype 1 and genotype 2 *env*. (**A**) Genome structure of the SFV YFP reporter viruses. The CMV immediate-early promoter (pCMV)-driven YFP (enhanced YFP, EYFP) expression cassette was inserted into the U3 region of the 3′ LTR, thereby deleting 709 bp of the LTR. The envelope gene of SFVmmu_R289hybAGM was inserted into this construct in order to generate two otherwise isogenic YFP reporter gene foamy virus proviral clones, AX571 pSFVmmu-DPZ9524_1_CMVie-YFP (ST1) and AX585 pDPZ9524_1_R289hybAGMenv_CMVie-YFP (ST2). The unspliced bel transcript designates the Tas mRNA, the spliced bel transcript designates the Bet mRNA. (**B**) Sequence alignment of the Env proteins of ST1 and ST2.

Quantification of infection directly via immunofluorescence or qPCR is laborious, expensive and does not allow for the analysis of a high number of samples as is necessary for the comparison of two different envelope genes under different conditions. We therefore generated a reporter virus based on that clone by inserting a CMV immediate-early promoter-driven *YFP* expression cassette into the U3 region of the 3′ LTR, thereby deleting 709 bp of the LTR. We chose this strategy after fusion of bet to mNeonGreen did not result in recoverable infectivity (not shown). This construct with the CMV promoter-driven *YFP* expression cassette (Figure 2A) yielded appreciable infectivity after transfection of 293T producer cells as evidenced by transduction of the *YFP* reporter gene into fresh 293T cells. The construct might technically be replication-competent as the U3 region contains a CMV promoter and no additional polyA site, and such foamy virus clones have been found to replicate in the form of replication-competent recombinant revertants from self-inactivating foamy virus vectors [45]; however, these recombinant viruses exhibited strongly reduced replication. In line with this previous report, we did not observe visible spread of the virus in culture after infection (not shown), indicating that the construct can be used as a single-cycle foamy virus for practical purposes, presumably because the *YFP* open reading frame disrupts the LTR and the additional *YFP* expression cassette after duplication of the LTR during reverse transcription might impact packaging. The envelope gene of SFVmmu_R289hybAGM, which differs considerably from that of SFVmmu-DPZ9524 in its amino acid sequence (Figure 2B) was inserted into this construct in order to generate two otherwise isogenic *YFP* reporter gene foamy virus proviral clones, AX571 pSFVmmu-DPZ9524_1_CMVie-YFP (ST1) and AX585 pDPZ9524_1_R289hybAGMenv_CMVie-YFP (ST2).

### 3.3. The SFVmmu_R289hybAGM env Gene Is Associated with Significantly Higher Cell-Cell Fusion Than That of SFVmmu-DPZ9524

**Figure 3:**
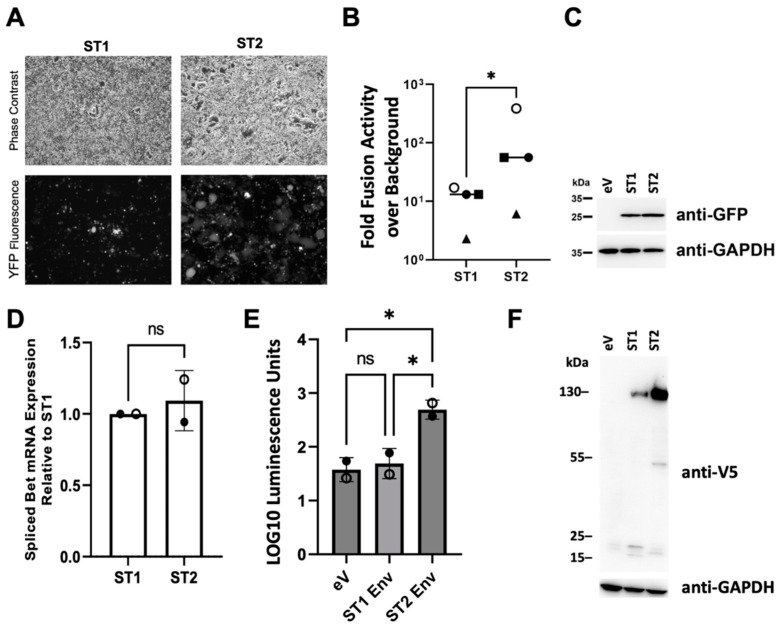
The R289hybAGM-derived *env* gene effects increased cell-cell fusion in the same genetic background. (**A**) Syncytia formation in 293T cells that were transfected with SFVmac ST1 and ST2 5 days post transfection. (**B**) Cell-cell fusion assay with ST1 and ST2 transfected 293T effector cells. The data show values normalized to background activity; (eV = empty vector). Four independent experiments were performed in triplicates. Luciferase activity in ST1-containing cocultures was significantly lower than in ST2-containing cocultures (*p* ≤ 0.05, *; paired *t*-test comparing the log-transformed fusion activity. Identical symbols represent mean values from the same experiment.). (**C**) Representative Western blot of ST1 and ST2 transfected effector cells. Expression of the YFP reporter gene product encoded by the two viruses was determined using anti-GFP antibody. GAPDH served as loading control. (**D**) Expression of the spliced Bet mRNA 24 h post transfection of 293T cells with ST1 or ST2 as measured by RT-qPCR. The data show expression normalized to ST1, averaged from two independent experiments (open and filled circles), each in biological triplicates. Error bars show the standard deviation. ns: non-significant, ratio paired *t*-test. (**E**) Fusion activity upon expression of ST1 and ST2 Env from their respective cDNAs under the control of the CMV immediate early promoter. The data show log10-transformed, averaged arbitrary luminescence units obtained in two independent experiments, one in biological triplicates, one in six biological replicates (open and filled circles represent mean values from the same experiment). eV = empty vector. *p* > 0.05, ns: non-significant; *p* ≤ 0.05, *; Ordinary one-way ANOVA. (**F**) Western blot analysis of lysates used for the determination of fusion activity in (**E**). The respective Env proteins were detected using a monoclonal anti-V5-epitope antibody recognizing the C-terminal V5-6His-tag.

Upon transfection of the two YFP reporter virus clones, we noticed strong syncytia formation with the SFVmmu_R289hybAGM Env-expressing ST2 construct, but less so with the original SFVmmu-DPZ9524 Env-expressing ST1 (Figure 3A). Therefore, we set up a fusion assay with reporter cells that were expressing a Gal4-driven TurboGFP-luciferase construct and effector cells transfected with ST1 or ST2 together with a VP16-Gal4 transactivator. These cells were mixed and cocultured, and luciferase activity was quantified as a readout for fusion. After mixing, in line with the increased syncytia formation visible upon transfection with ST2 compared to ST1, increased luciferase activity was observed with ST2-transfected effector cells (Figure 3B). Expression of YFP, which runs at a lower molecular weight than the TurboGFP-luciferase fusion protein (below detection limit, molecular weight approx. 85 kDa) and is encoded by ST1 and ST2, as detected by Western blot was comparable (Figure 3C). A change in *env* might affect the internal promoter that drives transcription of the unspliced Tas and the spliced Bet transcripts [46]. We therefore tested expression of the Bet transcript by RT-qPCR, which allows clear differentiation from genomic RNA/DNA or plasmid DNA through its splice site. We found no difference in expression at 24h post transfection (Figure 3D).

To ascertain that the observed difference in cell-cell fusion between ST1 and ST2 stems from the envelope protein itself, we also tested ST1 and ST2 Env in an isolated cell-cell fusion assay by expressing both proteins from their cloned cDNA sequences under the control of the cytomegalovirus immediate early promoter (Figure 3E). The ST2 Env-encoding plasmid in that assay drove readily detectable cell-cell fusion, whereas the ST1 Env-encoding plasmid was not significantly active over background levels. In Western blot analysis of these fusion reactions using the V5 tag fused to the C-terminus of each Env protein, ST2 Env was observed to be expressed to considerably higher levels, most likely indicative of higher synthesis, higher stability or a combination thereof (Figure 3F). In addition, the two proteins exhibited a different pattern of proteolytic fragments, suggestive of differential proteolytic processing.

### 3.4. Low Frequency of Cross-Neutralization between Sera Neutralizing Genotype 1 or Genotype 2

**Figure 4:**
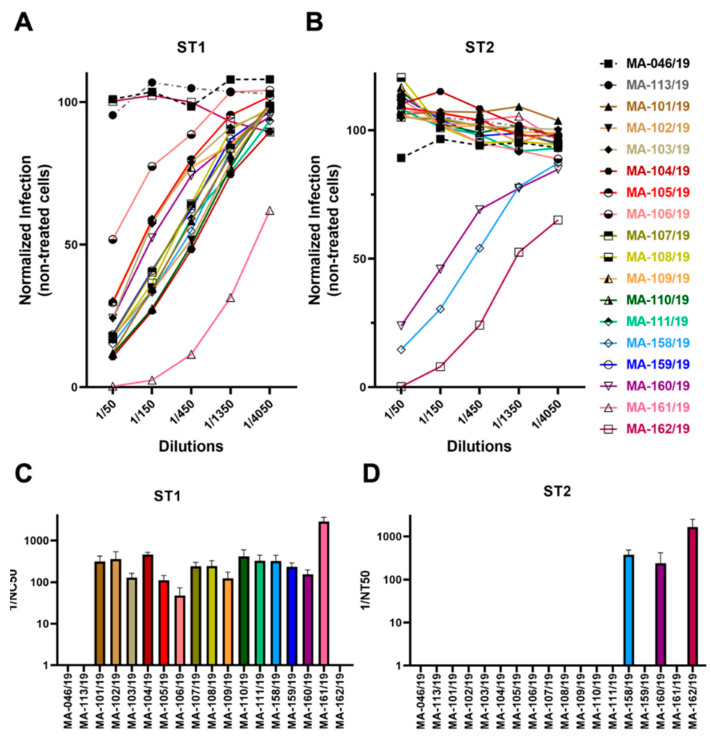
Low frequency of cross neutralization by sera from different animals. Neutralizing activity of rhesus macaque sera against ST1 (**A**) and ST2 (**B**). Infection was measured using flow cytometry to detect expression of the YFP reporter gene. The data show values normalized to infection without rhesus macaque serum. The experiment was performed twice with congruent results. Inverse neutralizing titers (1/NC50 values) were calculated for the individual rhesus macaque sera against ST1 (**C**) and ST2 (**D**) (nonlinear fit for different NC50, inhibitor vs. normalized response model). Error bars represent the 95% confidence interval.

We next tested whether the two Env proteins really belonged to different serological groups and whether they were resistant to neutralization by sera that neutralize the respective other genotype. We obtained 18 rhesus macaque sera, two of them negative for foamy virus according to prior serological testing [47]. Fifteen sera neutralized ST1 (Figure 4A,C) and three sera neutralized ST2 (Figure 4B,D). Only two sera, #158 and #160, neutralized both ST1 and ST2. At present, it is unclear whether this represents some degree of cross-neutralization or double infection of the animals. It should be noted that the two animals with sera neutralizing both ST1 and ST2 and the one neutralizing exclusively ST2, were housed together in the same group of animals (animals 158/19-162/19), compatible with infection with a genotype 2 SFV in that group of animals but not in all other animals, which form another group of animals with potential contact. Thirteen out of fifteen sera with neutralizing capacity against ST1 were without effect on ST2, arguing for at best minimal cross-neutralization between these two genotypes, at least in this direction.

### 3.5. No Evidence for env-Associated Differences in Cell Tropism

**Figure 5:**
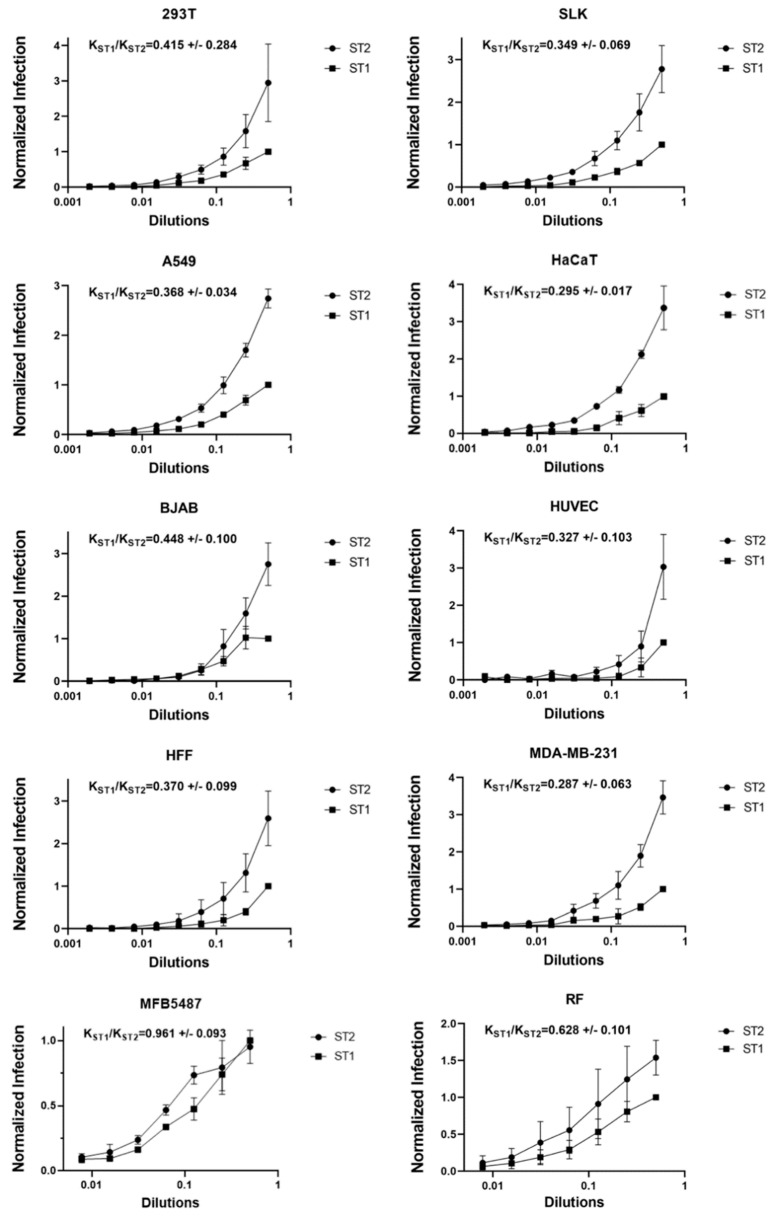
No evidence for *env*-associated differences in cell tropism. Infection of the indicated cells and cell lines after inoculation with increasing concentrations of ST1 or ST2. Infection was measured using flow cytometry to detect expression of YFP. The data show values normalized to infection with ST1 at the highest concentration. Curve fitting of infectivity on the different cell lines/cultures was performed using the built-in exponential equation for one phase association of GraphPad Prism version 9 to calculate K_ST1_ and K_ST2_ from the non-normalized data. The ratio between K_ST1_ and K_ST2_ represents the differences in infectivity between the viruses. Values represent the average of three experiments, error bars represent the standard deviation.

Tissue tropism of viruses is often determined by the interaction of their surface proteins with cellular receptors. The cellular receptors for foamy viruses so far remain unknown, even if heparan sulfate was described as an attachment factor [48]. Nevertheless, a putative receptor binding domain has been described [23], and the differences between genotypes 1 and 2 are predominantly found in that region of Env. We therefore hypothesized that the different *env* genes might confer different cellular tropism. To test this hypothesis, we infected several different cell lines and primary cells with ST1 and ST2 and compared the relative susceptibility of different cells to these two viruses (Figure 5). We consistently observed higher infectivity with ST2 than with ST1, even though both viruses were produced identically and in parallel. Nevertheless, as this was observed in all cells tested, the relative difference in infectivity between the two viruses, which was also calculated as ratio, was highly similar on all cells that were tested, indicating no major differences in tropism. In the one cell line where the two viruses seemed to perform more similarly (MFB5487), this was mostly due to 2 data points at the end of the dilution series and not consistently observed.

### 3.6. SFVmmu_R289hybAGM env Effects Lower Particle Release and Higher Specific Infectivity

**Figure 6:**
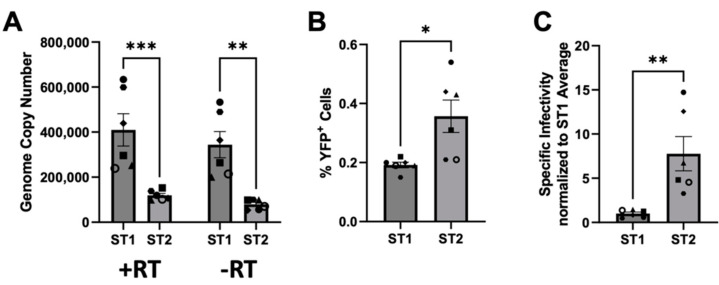
ST2 particles exhibit higher infectivity. (**A**) Viral genome copy number values of DNAse-treated supernatant of ST1 or ST2 producing 293T cells. Nucleic acid was extracted and analyzed by qPCR targeting the pol gene with or without prior reverse transcription (RT). Six biological replicates (rep) were performed. *p* ≤ 0.01, **; *p* ≤ 0.001, ***; 2-way ANOVA, Sidak’s correction for multiple comparisons. (**B**) The exact same supernatants as in (**A**) were used to infect fresh 293T indicator cells by spinfection. *p* ≤ 0.05, *; unpaired *t*-test. (**C**) Specific infectivity was calculated as the ratio of YFP-expressing cells over DNA genome copy number (-RT) for each sample and was normalized to the average ST1 infectivity. *p* ≤ 0.01, **; unpaired *t*-test. Error bars represent the standard error of the mean.

To follow up on the differences in infectivity between ST1 and ST2 preparations observed in Figure 5, we performed six independent transfections of ST1 and ST2 into 293T cells and first measured the release of DNAse-resistant viral nucleic acid by RT-qPCR and qPCR of the cell culture supernatants (Figure 6A). As observed previously [20,49], most of the foamy virus genome exists in the form of DNA within viral particles as evidenced by minimal differences in copy number values between PCR reactions with and without prior reverse transcription. Supernatant of ST2 producing cells contained on average less than half of the amount of viral nucleic acid as compared to supernatants from ST1 producing cells. Conversely, when these very same supernatants were tested for infectivity, ST2-containing supernatants produced approximately twice the number of YFP-positive cells (Figure 6B). Overall, when assuming the relationship of infectious particles to infected cells as linear for a multiplicity of infection below 0.01, specific infectivity of ST2 particles based on DNAse-resistant viral DNA (calculated individually for each sample from Figure 6A,B and averaged) was approximately 7.8 times higher than that of ST1 particles (Figure 6C).

### 3.7. No Evidence for env-Associated Differences in Susceptibility to IFITM-Mediated Restriction at the Cell Entry Step

**Figure 7:**
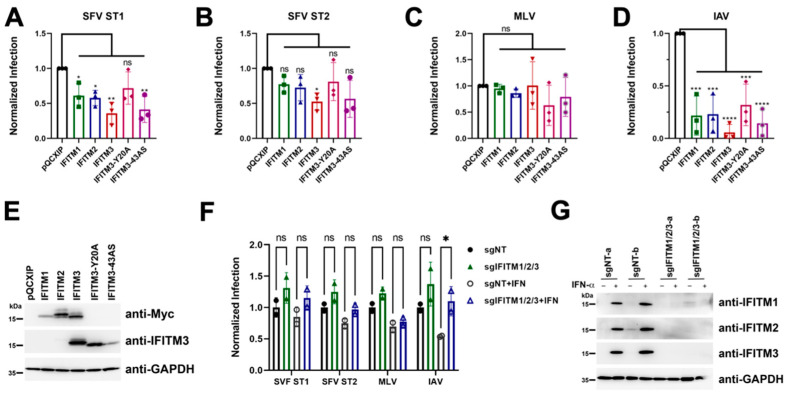
No evidence for *env*-associated differences in susceptibility to IFITM-mediated restriction. (**A–D**) A549 cells were transduced with pQCXIP-constructs to express IFITM1, IFITM2, IFITM3, IFITM3-Y20A, IFITM3-43AS or pQCXIP (empty vector). IFITM overexpressing cells were infected with SFV ST1, SFV ST2, MLV lentiviral pseudotype (MLV-LP) or IAV lentiviral pseudotype (IAV-LP). Infection was measured using flow cytometry to detect expression of the fluorescent reporter gene. The data show values normalized to pQCXIP (empty vector), and error bars represent standard error of the mean of five experiments, each performed in triplicates. Statistical significance was determined by ordinary one-way ANOVA (*p* > 0.05, ns; *p* ≤ 0.05, *; *p* ≤ 0.01, **; *p* ≤ 0.001, ***; *p* ≤ 0.0001, ****). (**E**) Representative Western blot of IFITM overexpressing cells. Expression of myc-tagged IFITMs was determined using anti-myc antibody. Expression of IFITM 3 was determined using anti-IFITM3 antibody. GAPDH served as loading control. (**F**) IFITM1/2/3 triple-knockout. A549 cells were transduced with lentiviral vectors encoding Cas9 and the sgRNAs IFITM-knockout (sgIFITM1/2/3-a, sgIFITM1/2/3-b) or control cells (sgNT-a, sgNT-b) treated with IFN-α (5000 U/mL) or H_2_O (control) and infected with SFV ST1, SFV ST2, MLV lentiviral pseudotype (MLV-LP) or IAV lentiviral pseudotype (IAV-LP). Infection was measured using flow cytometry to detect expression of the fluorescent reporter gene. The data show values normalized to the average of sgNT-a, sgNT-b and error bars represent standard error of the mean of five experiments, each performed in triplicates. Statistical significance was determined by 2-way ANOVA (*p* > 0.05, ns; *p* ≤ 0.05, *). (**G**) Representative Western blot of control (sgNT-a or sgNT-b) cells or IFITM knockout (sgIFITM1/2/3-a or sgIFITM1/2/3-b) treated with or without IFN-α (5000 U/mL). IFITM expression was detected with anti-IFITM1, anti-IFITM2 or anti-IFITM3; GAPDH served as loading control.

We next analyzed the susceptibility of genotype 1 and genotype 2 *env*-driven cell entry to restriction by IFITM expression. Very similar to our results regarding cellular tropism, there was no difference between the two *env* genes in that entry driven by either *env* gene product was susceptible to recombinant overexpression of IFITM proteins, which resulted in reduction in infection for overexpression of all IFITM proteins. The reduction in infection by both viruses was significant for IFITM3 (Figure 7A,B). For ST1, inhibition of infection by IFITM1, IFITM2 and the IFITM3 43AS mutant, which shows broader subcellular localization than IFITM3 [50], was significant (Figure 7A). Expression of the IFITM proteins was verified by Western blot (Figure 7E). The IFITM3 Y20A sorting motif mutant, also described to exhibit wider sub-cellular distribution [51], was slightly less potent than the 43AS mutant, hinting at subtle differences between these mutants and their effects. These results suggest that sufficiently high IFITM expression levels could at least in theory affect SFV infection. Comparison with lentiviral particles pseudotyped with either the IFITM-resistant murine leukemia virus (MLV) glycoprotein or the IFITM-sensitive influenza A virus glycoproteins (IAV), demonstrated that the effect of IFITM overexpression on foamy virus infection lies somewhere between the two, but the observed degree of inhibition was much less pronounced than that of IAV glycoprotein-driven infection.

We next tested the effect of IFITM knockout under conditions with and without prior induction of IFITM expression through treatment with IFN-α (Figure 7F). Simultaneous knockout of all three IFITM proteins in A549 cells resulted in a slight enhancement of infection for both ST1 and ST2, but this did not reach significance. It should also be noted that the enhancement was approximately 1.31 fold for ST1 under conditions without prior interferon stimulation, and this barely changed to 1.35 fold when the cells had been previously stimulated with interferon, with a similarly negligible change from 1.25 fold to 1.3 fold for ST2, despite strong induction of IFITM expression (Figure 7G). This is similar to observations with the MLV envelope-pseudotyped lentivirus, which was enhanced by 1.22 fold and 1.11 fold, respectively, upon IFITM knockout (Figure 7F). For comparison, the enhancement by IFITM knockout changed from 1.37 fold to 2.04 fold enhancement for influenza HA/NA-pseudotyped lentivirus upon interferon pretreatment (Figure 7F). These results strongly argue against appreciable effects of IFITMs at expression levels reached after interferon stimulation, at least in A549 cells. Further, neither of the two different *env* genes was associated with increased susceptibility to IFITM-mediated restriction in this assay.

## 4. Discussion

The SFVmmu-DPZ9524-based infectious clone and YFP reporter virus generated in this study (Figure 1 and Figure 2A) will facilitate further research on SFVs by adding to the available repertoire of viruses [19,52]. Using this system, we found to our surprise the genotype 2 *env* gene of SFVmmu_R289hybAGM endows SFVmmu-DPZ9524, originally a type 1 virus, with significantly higher fusion activity (Figure 3), and with higher infectivity across a range of target cells (Figure 5 and Figure 6), which was coupled with lower particle release (Figure 6A). Whether the higher fusion activity of the SFVmmu_R289hybAGM *env* gene product is due to higher expression levels, as suggested by our Western blot results, higher intrinsic fusogenicity, altered surface expression, different proteolytic processing or a combination of these factors is currently an open question. Nevertheless, our findings may be helpful for the construction of foamy virus-based gene vectors, as transduction efficiency is always an issue for viral vectors, and the SFVmmu_R289hybAGM envelope would clearly be a superior choice for such an application. Generally, our observations suggest that the different foamy virus envelope genes from genotypes circulating within a species may differ considerably with regard to their ability to mediate entry and cell-cell fusion, something so far only described for foamy virus *env* genes from different species [53]. As we only tested a single *env* from each genotype, these findings should not be generalized, and each one of the two tested *env* genes could represent an outlier or have acquired a particularly advantageous or deleterious mutation that may not be found in similar isolates from other animals. Nevertheless, our results suggest that foamy viruses may be able to quickly acquire enhanced fitness through acquisition of a different *env* gene through recombination, which would, in the case studied here, result in increased cell-cell fusion and at the same time release of higher absolute infectivity and higher per-particle infectivity. Given the known very high propensity to template switching of foamy viruses [54], the possibility of such recombination events should be kept in mind in light of the well-known zoonotic potential [9,10,11,12,13,52]. Frequent exchange of the variant parts of *env* has been proposed as one possible explanation for the high evolutionary stability of the foamy virus genome and its diversity in the *env* gene [22]. However, it is currently unclear how often such recombination events occur in co-infected animals.

Interestingly, our plasmid-derived infectious clone of SFVmmu-DPZ9524 replicated measurably better in primary rhesus monkey fibroblasts than the plasmid-derived SFVmmu_R289hybAGM clone (Figure 1), despite its comparatively suboptimal *env* gene. This suggests that other factors in these two viral clones may balance or, in this case, rather overcompensate the effects of *env*.

Our findings regarding neutralization clearly also confirm for these two rhesus macaque SFV *env* genes that the *env* genes cluster into genotypes that almost certainly have been selected for by the humoral immune response. All but two out of 15 genotype 1-neutralizing sera did not affect genotype 2 Env-driven infection (Figure 4B,D). This is similar to observations in an earlier report [55]. Only three sera neutralized ST2, and we found only one that exclusively neutralized ST2 and not ST1 (Figure 4A,C). Even so, we suspect double infection and not cross-neutralization for the two dual-reactive sera, as there was absolutely no cross-neutralization visible for the serum that exclusively neutralized ST2 and double infection was reported to be frequent in macaques [55]. The degree of resistance to cross-neutralization observed with most sera is remarkable in that the differences in the Env amino acid sequences lie mostly within the short, proposed receptor binding domain (Figure 2B). This demonstrates that large parts of the foamy envelope protein are not targeted by neutralizing antibodies during natural infection.

Our findings regarding IFITM-mediated restriction suggest that entry of macaque SFVs may be somewhat sensitive to IFITM3 if expressed at sufficiently high levels (Figure 7A,B), in line with an endosomal route of entry as reported for foamy viruses [53,56]. The observed sensitivity to inhibition by IFITMs was highly similar between ST1 and ST2. In light of the notion that IFITMs primarily restrict at the membrane fusion step [32,33], one might have expected that the more fusogenic ST2 would be more resistant to IFITM-mediated restriction. Nevertheless, this was not readily observed, even if ST1 may have exhibited slightly increased sensitivity to overexpression of individual IFITMs, but not in a meaningfully different manner. While convenient as an experimental system and important to establish whether individual IFITMs in principle possess the ability to restrict a pathogen, overexpression of individual IFITM genes by means of retroviral transduction may lead to aberrant localization and expression levels that exceed those normally found in interferon-stimulated cells [30]. In line with this notion, ST1 and ST2 infection was only minimally altered by simultaneous knockout of IFITMs 1-3 in the A549 cell line (Figure 7F), which is often used to study the effect of IFITM expression [30,33,51], and the effect did not reach significance, neither with nor without prior interferon induction of IFITM expression. In addition, the relative increase in infection was not profoundly different between these conditions, different from what was observed for IAV HA/NA-driven infection and different from what would be expected after strong, interferon-mediated induction of IFITM expression. While this manuscript underwent revision, IFITM3 was reported to restrict the entry of prototype foamy virus [57], in principle compatible with our findings in overexpression experiments testing the two macacine Env proteins. We focused our studies here on the entry process and did not analyze whether IFITMs act at a later stage of infection as shown for FFV [34] and now also for prototype foamy virus [57], and if at such a later stage there are differences between the two env genes. This might be a topic for future studies. At least for cells that are infected through entry pathways similar to those used for infection of A549, our results suggest that IFITM-mediated restriction may play a minor role in the control of macaque foamy virus infection at the cell entry step as compared to the IFITM-mediated restriction observed for IAV glycoprotein-driven entry.

## Figures and Tables

**Table 1:** Plasmids.

Plasmid	Source	Reference/Identifier
psPAX2	Addgene (kind gift from Didier Trono)	Addgene #12260
VSV-G (pMD2.G)	Addgene Addgene (kind gift from Didier Trono)	Addgene #12259
gag/pol	Addgene (kind gift from Tannishtha Reya)	Addgene #14887
pLenti CMV GFP Neo (657-2)	Lenti CMV GFP Neo (657-2) was a gift from Eric Campeau and Paul Kaufman	Addgene #17447 [38]
Gal4-TurboGFP-Luc (Gal4- driven TurboGFP-luciferase reporter plasmid)	Laboratory of Alexander Hahn	[39]
AX526 (Gal4-driven TurboGFP-luciferase reporter lentivirus, pLenti-Gal4-TurboGFP-Luc)	Laboratory of Alexander Hahn	[40]
plentiCRISPRv2 encoding sgRNAs targeting IFITM1-3 (sgIFITM1/2/3-a, sgIFITM1/2/3-b) or non-targeting sgRNAs (sgNT-a, sgNT-b)	lentiCRISRPv2 backbone from Addgene (kind gift from Feng Zhang), inserts from the laboratory of Alexander Hahn	[33,41]
Vp16-Gal4	Laboratory of Alexander Hahn	[39]
pCAGGS IAV_WSN-HA	Laboratory of Stefan Pöhlmann	[42]
pCAGGS IAV_WSN-NA	Laboratory of Stefan Pöhlmann	[42]
paMLV_env	Laboratory of Michael Farzan	[31]
pQXCIP	Laboratory of Stefan Pöhlmann	[42]
pQCXIP-IFITM1	Laboratory of Stefan Pöhlmann	[42]
pQCXIP-IFITM2	Laboratory of Stefan Pöhlmann	[42]
pQCXIP-IFITM3	Laboratory of Stefan Pöhlmann	[42]
pQCXIP-IFITM3-Y20A	Laboratory of Stefan Pöhlmann	[33]
pQCXIP-IFITM3-43AS	Laboratory of Stefan Pöhlmann	[33]

**Table 2:** Cell lines.

Cell Line/Cells	Cell Line Origin
HEK 293T	RRID:CVCL_0063, a kind gift from Vladan Rankovic, Göttingen, and originally purchased from the ATCC
A549	A kind gift from the laboratory of Stefan Pöhlmann, German Primate Center-Leibniz Institute for Primate Research, Göttingen, Germany
SLK	RRID:CVCL_9569, NIH AIDS Research and Reference Reagent program
Human foreskin fibroblasts (HFF)	A kind gift from the laboratory of Klaus Korn, Universitätsklinikum Erlangen, Institute for Clinical and Molecular Virology, Erlangen, Germany
Human vascular endothelial cells (HUVEC)	PromoCell
HaCaT	RRID:CVCL_0038, CLS Cell Lines Service GmbH
MDA-MB-231	ATCC HTB-26, a kind gift from the laboratory of Felix Engel
BJAB	Leibniz-Institute DSMZ (Deutsche Sammlung von Mikroorganismen und Zellkulturen GmbH)
MFB5487	*Macaca fuscata* B cell line, established from PBMC through immortalization with Herpesvirus papio. A kind gift from Ulrike Sauermann [40]
Primary rhesus monkey fibroblasts	A kind gift from the laboratory of Rüdiger Behr.

**Table 3:** Antibodies.

Target	Details
IFITM1	Goat, R&D (AF4827), 1:500 (secondary: Proteintech rabbit anti-goat 1:10,000)
IFITM2	Mouse, Proteintech (66137-1-lg), 1:1000 (secondary: Dianova donkey anti-mouse 1:10,000)
IFITM3	Rabbit, Cell Signal Technology (D8E8G) 1:1000 (secondary: Life Technologies goat anti-rabbit 1:10,000)
c-Myc	Mouse, Santa Cruz Biotechnology (9E10) 1:1000 (secondary: Dianova donkey anti-mouse 1:10,000)
V5	Mouse, BioRad (MCA1360), 1:1000 (secondary: Dianova donkey anti-mouse 1:10,000)
GAPDH	Mouse, GenScript, 1:5000 (secondary: Dianova donkey anti-mouse 1:10,000)
GFP	Rabbit, GenScript, 1:1000 (secondary: Life Technologies goat anti-rabbit 1:10,000)

## Data Availability

Not applicable.

## Supplementary Materials

The following supporting information can be downloaded at: https://www.mdpi.com/article/10.3390/v15020262/s1, Table S1: Nucleotide variants.
